# Presentation of the Child with Renal Disease and Guidelines for Referral to the Pediatric Nephrologist

**DOI:** 10.1155/2012/978673

**Published:** 2012-05-28

**Authors:** Amin J. Barakat

**Affiliations:** Department of Pediatrics, Georgetown University Medical Center, Washington, DC 20007, USA

## Abstract

Renal disease is a major cause of morbidity and mortality. Pediatric patients with renal disease, especially younger ones may present with nonspecific signs and symptoms unrelated to the urinary tract. Pediatricians, therefore, should be familiar with the modes of presentation of renal disease and should have a high index of suspicion of these conditions. Affected patients may present with signs and symptoms of the disease, abnormal urinalysis, urinary tract infection, electrolyte and acid-base abnormalities, decreased renal function, renal involvement in systemic disease, glomerular and renal tubular diseases, congenital abnormalities, and hypertension. Pediatricians may initiate evaluation of renal disease to the extent that they feel comfortable with. The role of the pediatrician in the management of the child with renal disease and guidelines for patient referral to the pediatric nephrologist are presented.

## 1. Introduction

 Renal disease is a major cause of morbidity and mortality [1–3]. Pediatric patients especially younger ones with renal disease may present with nonspecific signs and symptoms unrelated to the urinary tract. Pediatricians, therefore, should be familiar with the modes of presentation of different renal conditions and should have a high index of suspicion of renal disease. Early diagnosis and treatment of renal disease in children is important in the prevention of renal failure and end-stage renal disease (ESRD). I will discuss here the presentation of the child with these diseases and outline guidelines for patient referral to the pediatric nephrologist. 

## 2. Presentation of the Child with Renal Disease

 Patients with renal disease may present with (1) signs and symptoms of renal disease, (2) abnormal urinalysis, (3) urinary tract infection (UTI), (4) electrolyte and acid-base abnormalities, (5) decreased renal function, (6) renal involvement in systemic disease, (7) glomerular disease, (8) renal tubular disease, (9) congenital abnormalities of the kidney or urinary tract, and (10) hypertension (HT). Often, renal disease may be asymptomatic; therefore, a blood pressure determination, a thorough abdominal examination, and a urinalysis should be an integral part of a routine medical examination in children.

## 3. Signs and Symptoms of Renal Disease

 Renal disease, particularly in children, may present in a subtle manner such as failure to thrive, unexplained fevers, vague pains, gastrointestinal symptoms, anemia, abdominal mass, edema, HT, and metabolic acidosis. Failure to thrive may suggest chronic kidney disease or renal tubular disease. Anemia, growth failure, HT, and abnormal retinal changes may be the first signs of chronic kidney disease. Frequency, urgency, dysuria, hesitancy, and urinary retention suggest UTI, obstructive uropathy, or urinary calculi.

 Physicians should be familiar with the normal voiding pattern of children at various ages. *Frequency* is frequent urination suggesting UTI, while *polyuria* is the passage of a larger amount of urine than normal. It indicates decrease in concentrating ability which occurs in diabetes mellitus, diabetes insipidus, chronic pyelonephritis, or chronic kidney disease. *Pollakiuria* (Greek pollakis, meaning often) is a common symptom affecting toilet-trained school children especially boys. It refers to daytime isolated urinary frequency which has a sudden onset and lasts from a few days to a few weeks. Affected children have a normal physical examination, urinalysis and urine culture, and do not require further investigation.

*Enuresis *(nocturnal incontinence) is bedwetting beyond the age when the child should be able to control urination. It is usually idiopathic and associated with a positive family history. It initially requires no other investigation than a urinalysis and urine culture. Secondary and diurnal forms of enuresis, as well as enuresis beyond the age of 12 years, may require urologic evaluation. *Nocturia* in older children is defined as awakening at night to pass urine. This may be normal, or may suggest a decrease in urine concentrating ability and may also be an early sign of chronic kidney disease.

It is important to keep in mind that renal disease including UTI in children may present in a subtle manner. Physicians, therefore, should have a high index of suspicion and should perform urinalyses and urine cultures on any child with unexplained fevers.

Most renal diseases are painless. Acute pyelonephritis, renal calculi, and trauma to the kidney or bladder may present with abdominal or flank pain. *Dysuria*, or pain on urination, is a symptom of UTI or urethritis. The pain of cystitis or prostatitis is usually suprapubic and gradual in onset.

Abdominal masses of renal origin may represent hydronephrosis, multicystic, dysplastic or polycystic kidney disease, renal vein thrombosis, and Wilms tumor or neuroblastoma.

## 4. Abnormal Urinalysis

 Patients with kidney disease may present with abnormal urinary findings. A carefully performed urinalysis using physical, chemical, and microscopic examination is an easy and informative tool to the practicing physician [4]. The American Academy of Pediatrics recommends a urinalysis as a part of preventive pediatric health care at age 5 years and mid-adolescence [5]. An abnormal urinalysis may be the only presenting sign of chronic GN.

 The most common urinary abnormalities are hematuria and proteinuria. *Hematuria* may be gross or microscopic, discovered during a routine urinalysis. Evaluation of the child with hematuria may be easily initiated by the primary care physician. Urinalysis and, when indicated, an audiogram on immediate family members should be performed, since recurrent benign hematuria, Alport syndrome, IgA nephropathy, and other forms of glomerular disease may be familial. In general, the presence of persistent and recurrent gross hematuria should prompt referral to a pediatric nephrologist. 

 Persistent *proteinuria *should be investigated. The primary care physician may quantitate the proteinuria and exclude the orthostatic type. Significant proteinuria (>1 g/1.73 m^2^/day), or proteinuria associated with abnormal RBC morphology, decreased renal function, HT, low serum complement, or manifestations of systemic disease are suggestive of glomerular disease and are indications for renal biopsy.

*Pyuria* may originate from any part of the urinary tract and usually suggests UTI, but it may be seen also with any inflammatory process of the kidney and urinary tract, renal calculi and abnormalities of the urinary tract. *Casts* are of diagnostic importance. Red blood cell casts, for example indicate glomerular bleeding. 

## 5. Urinary Tract Infection

 Urinary tract infection (UTI) is the most common bacterial disease responsible for long term morbidity in children [6]. Accurate and prompt diagnosis and treatment are crucial and may prevent renal scarring. Diagnosis of UTI requires a high degree of suspicion because of the nonspecific nature of symptoms in younger children such as unexplained fevers, gastrointestinal symptoms, and irritability. The diagnosis is established by a quantitative urine culture. Because of the high association of UTI with vesicoureteral reflux and other urinary tract abnormalities, imaging studies should be considered.

## 6. Electrolyte and Acid-Base Abnormalities

 Electrolyte and acid-base abnormalities are commonly seen in pediatric practice. Patients may present with nausea, vomiting, diarrhea, decreased intake of fluids, irritability, lethargy, weight loss, dry skin and mucus membranes, elevated pulse, seizures and coma. The most common cause of acid-base disorder in children is metabolic acidosis secondary to diarrheal dehydration; however, affected children may present with a very complex clinical picture, and treating physicians should be familiar with the intricacies of their diagnosis and management. Severely affected patients should be referred immediately to a hospital where expert care can be delivered.

## 7. Decreased Renal Function

*Azotemia* is elevated serum urea nitrogen, *renal failure* is reduction in renal function, and *uremia* is the syndrome that encompasses the overt consequences of chronic kidney disease such as anemia, osteodystrophy, and central nervous system, gastrointestinal and other manifestations. *Acute kidney injury* is an abrupt severe reduction in glomerular filtration and is characterized by oliguria (urine < 0.5 mL/kg/hr) or anuria. The etiology of acute kidney injury should be identified promptly because many causes are reversible, and because management and prognosis of this condition vary with the specific etiology. Affected patients should be referred to a pediatric nephrologist immediately.

 The presence of growth retardation, anemia, history of underlying renal disease, renal osteodystrophy or small, contracted kidneys suggests the presence of *chronic kidney disease*, which is defined as the stage at which the kidneys are irreversibly damaged and unable to maintain the body homeostasis. Congenital renal abnormalities of the urinary tract are the most prevalent cause of chronic kidney disease in young children, whereas GN is more prevalent in adolescents [7]. These patients should also be referred to the pediatric nephrologist, since they frequently progress to ESRD, requiring chronic dialysis and renal transplantation. Since many causes of ESRD in children are potentially preventable (hereditary and congenital abnormalities of the kidney and UTI), early diagnosis and treatment of these conditions is of utmost importance.

## 8. Renal Involvement in Systemic Diseases

 Various systemic diseases (systemic vasculitis-systemic lupus erythematosis, Henoch-Schönlein purpura, hemolytic uremic syndrome, sickle cell disease, and malignancy) and syndromes (chromosomal aberrations, Rubinstein-Taybi, Cornelia de Lange, and many others) may affect the kidney in childhood [1]. Renal involvement should be excluded in any individual with multisystem disease (collagen disease, diabetes mellitus, and storage diseases). Systemic diseases associated with glomerular abnormalities may present with arthritis, rash, hypertension, hematuria, or proteinuria. The diagnosis of renal involvement in systemic disease is based on clinical findings (hematuria, proteinuria, hypertension, and decreased serum complement levels, decreased renal function) as well as renal histology.

## 9. Glomerular Disease

 The majority of children with glomerulonephritis (GN) present with proteinuria, hematuria, hypertension, edema, reduced renal function, or the nephrotic syndrome. Poststreptococcal acute GN is familiar to the practicing pediatrician. Most affected children have a benign course and can be easily treated by the primary care physician on an ambulatory basis. Obviously, a nephrology consultation should be obtained on patients with oliguria, hyperkalemia, nephrotic syndrome, cardiac overload, and renal insufficiency. Patients with prolonged oligoanuria, a persistently low serum complement for more than 8 weeks, or associated nephrotic syndrome may require a kidney biopsy. 

Nephrotic syndrome is characterized by proteinuria ≥ 40 mg/m^2^/hr (or 50 mg/kg/day), serum albumin < 2.5 g/dL and variable degrees of edema. The most common form of nephrotic syndrome in children is *minimal change nephrotic syndrome*, which is characterized by response to corticosteroids and good prognosis, although most patients have one or more relapses. Patients with this type of nephrosis may be treated by the primary care physician, while those who are steroid-resistant or -dependent, those with a suspected structural glomerular abnormality, and those associated with systemic disease should be referred to the pediatric nephrologist, since they usually require a kidney biopsy, knowledge of the current therapeutic regimens, and a close followup.

## 10. Renal Tubular Disease

 Renal tubular diseases (renal glucosuria, Fanconi syndrome with or without cystinosis, aminoacidurias, renal tubular acidosis, nephrogenic diabetes insipidus, and others) are rare and complex, and their management usually requires the help of a pediatric nephrologist [8]. Affected patients may present with failure to thrive, acidosis, glucosuria, aminoaciduria, phosphaturia, rickets, and inability to concentrate the urine. Renal tubular acidosis should be considered in patients with metabolic acidosis and persistently alkaline urine. A positive family history may suggest the presence of these conditions.

## 11. Congenital Abnormalities of the Kidney and Urinary Tract

 Congenital abnormalities of the kidney and urinary tract are reported to occur in 5 to 10% of the population [9]. They represent 25% of the total ultrasonographically diagnosed malformations that occur in 0.25–0.7% of fetuses. About 1/3 to 2/3 of ESRD in children are due to congenital abnormalities of the kidney and urinary tract. In addition, these abnormalities occur in 23% of patients with chromosomal aberrations, and 2/3 of patients with abnormalities of other organ systems. Some of these abnormalities are minor and are discovered incidentally; others are major, leading to obstruction, renal scarring, pyelonephritis, and ESRD. Urinary tract abnormalities should be suspected in any child with UTI, congenital anomalies of other organ systems (cardiovascular, gastrointestinal, central nervous system, and others), chromosomal aberrations, various malformation syndromes, and those with single umbilical artery or supernumerary nipples. Prenatal diagnosis of these conditions by ultrasonography as early as 12–16 weeks gestation will reduce the occurrence of renal damage and ESRD.

## 12. Hypertension

 The prevalence of hypertension in children ranges from less than 1% to 5.1% [10]. While pediatric hypertension was previously assumed to be secondary to renal, cardiovascular or endocrine causes, there is now increased evidence that it could be a part of a spectrum of essential hypertension, mainly linked to the obesity epidemic. The three most common symptoms of hypertension in children are headache, difficulty sleeping, and tiredness, all of which improve with treatment. Pediatricians can play a pivotal role in the early diagnosis and treatment of HT to reduce long-term cardiovascular morbidity and mortality. Blood pressure should be measured routinely in every child starting at age three years and in children with comorbid conditions such as the presence of heart or kidney disease, obesity, history of umbilical line, or UTI [11]. Referral to a specialist depends on the level of comfort of the pediatrician and the degree of the etiological complexity.

## 13. Guidelines for Patient Referral

In the present era of managed care, primary care physicians find themselves performing some duties that have traditionally been performed by the specialist. It is difficult to clearly delineate indications for referral of patients to the pediatric nephrologist. In general, pediatricians may initiate evaluation to the extent that they feel comfortable with. The most common reasons for referral to a pediatric nephrologist include fluid and electrolyte disorders and hematuria/proteinuria, followed by chronic GN, nephrotic syndrome, UTI, hypertension, acute GN, and ESRD [12].

The role of the pediatrician in the management of the child with renal disease is outlined in Table 1. Guidelines for patient referral to the pediatric nephrologist are listed in Table 2.

## Figures and Tables

**Table 1:** Role of the pediatrician in the management of the child with renal disease [1].

(1) keep a high index of suspicion for UTI and renal disease
(2) take patient/family history, perform a complete physical exam with BP, and exclude the presence of systemic diseases
(3) perform a urinalysis on patient, and, when indicated, on family members, urine culture, antibiogram, and other laboratory tests: BUN, creatinine, electrolytes, serum complement, quantitative proteinuria, and creatinine clearance
(4) order imaging studies: renal ultrasound, VCUG, renal scan and others on patients with UTI, and suspected congenital abnormalities and calculi
(5) screen for orthostatic proteinuria and tubular disorders
(6) treat UTI, uncomplicated acute GN, conditions not associated with acute or progressive deterioration of renal function: minimal change nephrotic syndrome, mild abnormalities and others that the physician is comfortable with
(7) follow-up patients that the physician is comfortable with
(8) discuss and refer children with renal and urinary tract abnormalities diagnosed on routine prenatal ultrasound

UTI: urinary tract infection; BP: blood pressure; BUN: blood urea nitrogen; VCUG: voiding cystourethrogram; GN: glomerulonephritis.

**Table 2:** Guidelines for patient referral to the pediatric nephrologist [1].

(1) persistent unexplained hematuria, nonorthostatic proteinuria and HT
(2) decreased renal function (acute, chronic, and ESRD)
(3) renal tubular disease
(4) nephrotic syndrome, particularly steroid-dependent or -resistant
(5) atypical or persistent GN
(6) unexplained and severe acid-base and electrolyte abnormalities
(7) systemic diseases associated with progressive renal involvement-systemic SLE and diabetes mellitus
(8) genetic and congenital abnormalities likely to produce progressive renal damage
(9) when invasive studies, for example, kidney biopsy, are indicated
(10) major renal/urinary tract abnormalities found on routine prenatal ultrasound
(11) renal disease that is likely to progress—FSGN and IgA nephropathy
(12) conditions associated with acute complications—HT, calculi, and HUS
(13) when teamwork is needed—urologist, geneticist, dietician, and social worker
(14) parental anxiety

HT: hypertension; ESRD: end-stage renal disease; GN: glomerulonephritis; SLE: systemic lupus erythematosis, FGS: focal glomerulosclerosis; HUS: hemolytic uremic syndrome.

## Abbreviations

ESRD: End-stage renal disease
GN: Glomerulonephritis
HT: Hypertension
UTI: Urinary tract infection.
